# Diagnostic Efficacy of Voxel-Mirrored Homotopic Connectivity in Vascular Dementia as Compared to Alzheimer’s Related Neurodegenerative Diseases—A Resting State fMRI Study

**DOI:** 10.3390/life11101108

**Published:** 2021-10-19

**Authors:** Eva Y. W. Cheung, Y. F. Shea, Patrick K. C. Chiu, Joseph S. K. Kwan, Henry K. F. Mak

**Affiliations:** 1 Department of Diagnostic Radiology, LKS Faculty of Medicine, The University of Hong Kong, Hong Kong, China; cheungev@connect.hku.hk; 2 School of Medical and Health Sciences, Tung Wah College, Hong Kong, China; 3 Division of Geriatrics, Department of Medicine, Queen Mary Hospital, Hong Kong, China; syf321@ha.org.hk (Y.F.S.); chiukc@ha.org.hk (P.K.C.C.); 4 Department of Brain Sciences, Imperial College London, London W12 0NN, UK; jskkwan@hku.hk; 5 State Key Laboratory of Brain and Cognitive Sciences, The University of Hong Kong, Hong Kong, China; 6 Alzheimer’s Disease Research Network, The University of Hong Kong, Hong Kong, China

**Keywords:** resting state functional MRI, voxel-mirrored homotopic connectivity, functional connectivity, Alzheimer’s disease, mild cognitive impairment, vascular dementia

## Abstract

Previous studies have demonstrated that functional connectivity (FC) of different brain regions in resting state function MRI were abnormal in patients suffering from mild cognitive impairment (MCI) and Alzheimer’s disease (AD) when comparing to healthy controls (HC) using seed based, independent component analysis (ICA) or small world network techniques. A new technique called voxel-mirrored homotopic connectivity (VMHC) was used in the current study to evaluate the value of interhemispheric functional connectivity (IFC) as a diagnostic tool to differentiate vascular dementia (VD) from other Alzheimer’s related neurodegenerative diseases. Eighty-three participants were recruited from the university hospital memory clinic. A multidisciplinary panel formed by a neuroradiologist and two geriatricians classified the participants into VD (13), AD (16), MCI (29), and HC (25) based on clinical history, Montreal Cognitive Assessment Hong Kong version (HK-MoCA) neuropsychological score, structural MRI, MR perfusion, and 18-F Flutametamol (amyloid) PET-CT findings of individual subjects. We adopted the calculation method used by Kelly et al. (2011) and Zuo et al. (2010) in obtaining VMHC maps. Specific patterns of VMHC maps were obtained for VD, AD, and MCI to HC comparison. VD showed significant reduction in VMHC in frontal orbital gyrus and gyrus rectus. Increased VMHC was observed in default mode network (DMN), executive control network (ECN), and the remaining salient network (SN) regions. AD showed a reduction of IFC in all DMN, ECN, and SN regions; whereas MCI showed VMHC reduction in vSN, and increased VMHC in DMN and ECN. When combining VMHC values of relevant brain regions, the accuracy was improved to 87%, 92%, and 83% for VD, AD, and MCI from HC, respectively, in receiver operating characteristic (ROC) analysis. Through studying the VMHC maps and using VMHC values in relevant brain regions, VMHC can be considered as a reliable diagnostic tool for VD, AD, and MCI from HC.

## 1. Introduction

With the aging of the world population, dementia cases increase rapidly due to neurodegenerative diseases. In 2016, 47 million people suffered from dementia globally. It is projected to be more than 131 million in 2050. There are several types of dementia, the most common type is Alzheimer’s disease (AD), which contributes to 60% of dementia [1]. Although the pathophysiology of AD has not been completely conclusive, research has suggested that both amyloid and tau accumulation in brain are involved in driving the disease. Mild cognitive impairment (MCI) is the prodromal stage of AD and its conversion rate to AD is 10–15% per year [2]. Vascular dementia (VD) is the second most common form of dementia (20%), which is associated with the cerebrovascular conditions, such as a series of small strokes. The symptom of VD is subtle and temporary, and it is difficult to be detected [3]. Current neuroimaging techniques for dementia diagnosis mainly focus on detection of brain structural and functional changes: structural MRI to detect hippocampal atrophy and its correlation with progression of disease from MCI to AD [4,5,6,7]; Positron Emission Tomography imaging (PET) using 18F-FDG as tracer to detect metabolic reduction in AD and MCI [8]; 18F-Flumetametamol as tracer to detect amyloid deposition for AD [9,10,11]. For VD, structural imaging is the main technique to detect silent brain infarcts and medial temporal lobe atrophy [12,13]. Fluid-attenuated inversion recovery (FLAIR) MRI and diffusion weighted (DW) MRI are employed for detecting white matter change, lacunar infarcts, and white matter damage, respectively. However, these identifications are similar to stroke or white matter disease, which is not specific to VD [14,15]. 

Resting state functional MRI (rs-fMRI) is an upstream imaging technique, which is sensitive to the subtle change in brain functions. Voxel-mirrored homotopic connectivity (VMHC) is one of the rs-fMRI analysis methods that correlates functional connectivity between each voxel in one hemisphere and its corresponding voxel in the opposite hemisphere [16]. The voxel-wise interhemispheric coordination that dementia patients usually suffered can be demonstrated. In initial stage, VMHC has been applied to several neuropsychiatric disorders [17,18,19,20,21,22]. For dementia, different VMHC patterns have been found in Alzheimer’s disease (AD) and mild cognitive impairment (MCI) when compared to normal healthy controls (HC) [23,24]. However, the application in VD has not been well discussed.

Many studies have demonstrated aberrant functional connectivity of different brain regions in terms of brain networks, including default mode network (DMN), salient network (SN), and executive control network (ECN) in patients that suffered from dementia. These networks are thought to provide a general framework of human normal functioning. Aberrant functional connectivity within these networks could be used as a high sensitivity biomarker to characterize different types of dementia [25,26]. 

In this study, we compared the interhemispheric resting-state functional connectivity (IFC) of cognitively impaired subjects and healthy controls (HC) using VMHC analysis. We hypothesized that the IFC of VD patients could be aberrant when compared to healthy controls. The specific pattern of the VMHC map and VMHC values in different regions or networks can be used as biomarkers for early diagnosis of VD, as compared to Alzheimer’s related neurodegenerative diseases.

## 2. Method

### 2.1. Participants

As described in our previous study [27], dementia or cognitive impaired subjects were referred by the geriatricians from the memory clinic of the Queen Mary Hospital, Hong Kong for a prospective study during June 2017 to June 2019. A multi-disciplinary panel with two geriatricians (Y.F.S., P.K.C.C.) and a neuroradiologist (H.K.F.M) confirmed the final diagnosis for each subject, with the support from the findings from clinical (baseline and follow-up), neuropsychological assessment, i.e., Montreal Cognitive Assessment—Hong Kong version (HK-MoCA), and neuroimaging (structural MRI, PCASL-MR perfusion, MRA, and amyloid PET-CT). The diagnostic criteria were detailed in the prior study [27].

The final diagnosis of the current cohort included 16 Alzheimer’s disease, 29 mild cognitive decline, and 13 vascular dementia. Twenty-five healthy elderlies were recruited from local community centers as healthy controls. Details are listed in Table 1. Participants with the following conditions were excluded: was in active infection, or organ failure at the time of scan; was a drug abuser or a regular alcohol drinker; nonambulatory; had a history of seizures; migraine; psychiatric diseases; head injury; stroke; cancer within 5 years. Written consent was obtained from each participant. The study logistics complied with the Declaration of Helsinki. Ethics approval was obtained from the Institutional Review Board of the University of Hong Kong/Hospital Authority Hong Kong West Cluster (IRB reference: UW11-126). 

### 2.2. Clinical and Neuropsychological Assessment

Vascular risk factors (including hypertension, hyperlipidemia and diabetes) of all participants were obtained, either from an interview conducted by a nurse, or from medical record retrieved from the hospital clinical management system database. All participants underwent HK-MoCA which was administered by a trained research assistant [28]. 

### 2.3. Data Acquisition

#### 2.3.1. T1W Images

MRI images were acquired using a Philips Achieva 3T equipped with 32-channel phased-array head coil (Department of Diagnostic Radiology, the University of Hong Kong). A T1W MPRAGE (magnetization prepared rapid acquisition gradient-echo): repetition time [TR] = 6.75 ms, echo time [TE] = 3.163 ms, inversion time [TI] = 844 ms, flip angle = 8°, slice thickness = 1.2 mm, 256 sagittal slices; acquisition matrix = 256 × 256, field of view = 256 × 256 mm^2^, voxel size = 1 × 1 × 1.2 mm^3^, band width = 241 Hz/pixel) was obtained.

#### 2.3.2. Resting State Functional Images

Each participant was asked to remain quiet and relax during the scan, with their eyes closed but not to fall asleep. Resting state functional MRI (rs-fMRI) images were obtained with following parameters (multi-echo echo planar imaging (EPI) sequence; 180 time points; TR = 2000 ms, TE = 30 ms, flip angle = 90°, slice thickness = 4 mm, FOV = 230 × 230 mm^2^, acquisition matrix = 72 × 72, 36 slices, voxel size = 1.6 × 1.6 × 4 mm^3^). 

#### 2.3.3. Pre-Processing of Resting State Functional Images

Data pre-processing was conducted by Data Processing Assistant for Resting State fMRI (DPARSF) v.4.4 (http://www.rfmri.org/dparsf, accessed on 9 October 2021) with Statistical Parametric Mapping software (SPM12 http://www.fil.ion.ucl.ac.uk/spm/software/spm12/, accessed on 9 October 2021) on a MATLAB platform 2018a (The MathWorks, Inc., Natick, MA, USA). Preprocessing was performed with standard processing steps suggested by the developer of DPARSF [29], with details as follows: the first 10 images volume from each subject was discarded to eliminate magnetic saturation effects, and the remaining 170 images were re-sliced for time and motion correction (the 35th slice was used as reference). A maximum displacement in any direction (x, y, and z) of 2 mm and maximum rotation (x, y, and z) of 2 degrees during the rs-fMRI scan were limited. To spatially normalize the fMRI image, the high-resolution individual T1-weighted images were co-registered to the mean fMRI; the resulting aligned images were segmented and transformed into Montreal Neurological Institute (MNI) space using the DARTEL toolbox, and a group template was generated. Then, the motion-corrected functional images were specifically normalized to the group template using the transfer parameter estimated through DARTEL segmentation and resampled to voxel size of 3 × 3 × 3 mm^3^. The fMRI images were smoothed with Gaussian kernel of 4 × 4 × 4 mm^3^ full width half maximum (FWHM). To minimize low-frequency drift and high-frequency physiological noise, temporal band-pass filtering (0.01–0.1 Hz) was applied. Frame-wise displacement (FD) threshold of 0.5 was applied to reduce the motion artefacts, where bad points were interpolated using the nearest neighbor algorithm. Finally, a nuisance linear regression was performed with the white matter, cerebrospinal fluid, global signal, six head motion parameters at one time point earlier, and the 12 corresponding squared items (Friston 24-parameter model) as covariates.

#### 2.3.4. Voxel-Mirrored Homotopic Connectivity VMHC

For VMHC, pre-processed images were transformed to a symmetric brain template with the application of an rs-fMRI image. The template was obtained by flipping the left or right hemispheres along the X-axis midline and average with the original image. From each participant, the T1w and rs-fMRI images were normalized to the MNI space and co-registered to this group-specific symmetric template. The interhemispheric functional connectivity was analyzed based on the Pearson’s correlation between paired voxels located in exactly the same location but in a different hemisphere. The resultant correlations composed the VMHC map, the VMHC value of a particular region of interest (ROI) could be extracted from the corresponding VMHC map for further comparison. All the results were presented using the unilateral (left side only) brain mask, due to the fact that VMHC values were symmetric. VMHC values of DMN, ECN, SN and supplementary motor area, a total of 36 regions, were recorded. 

#### 2.3.5. Leukoaraiosis and Brain Regional Volume Segmentation

Leukoaraiosis, also called white matter hyper-intensity, was described as subcortical hyper-intensity lesions in the white matter, either diffuse in periventricular regions, or multifocal [30]. It can be shown in T2W FLAIR image as hyper-intensity. Wei et al. (2019) showed that white matter hyper-intensities from T2-FLAIR is equivalent to the while matter hypo-intensities derived from T1W MRI by FreeSurfer [31]. In the current study, brain regional volumes and leukoaraiosis volume were calculated from T1W MRI using the FreeSurfer software package (http://surfer.nmr.mgh.harvard.edu/ ver7.1, accessed on 10 October 2021) [32]. The subcortical volume segmentation and measurement of different brain structure were done by automated procedures [33]. Briefly, the process contained motion correction, removal of non-brain tissue using a hybrid watershed/surface deformation procedure [34] automated Talairach transformation, segmentation of the subcortical white matter and deep gray matter volumetric structures (including the hippocampus, amygdala, caudate, putamen, and ventricles) [33,34], intensity normalization, tessellation of the gray-white matter boundary, automated topology correction [35,36], and surface deformation following intensity gradients to optimally place the gray-white matter and gray matter/CSF borders at the location where the greatest shift in intensity defines the transition to the other tissue class [37,38,39]. 

### 2.4. Statistical Analysis 

Demographic data including age, gender, HK-MoCA scores between AD, VD, MCI, and HC groups were analyzed using one-way ANOVA on rank test with post-hoc Bonferroni test. *p* < 0.05 was considered to be significantly different in all tests. Analyses were performed using SPSS software version 25.0 (SPSS Inc., Chicago, IL, USA). VMHC value between different groups was examined in a voxel-wise manner based on the DPARSF software. A two sample independent t-test was performed to compare the participant images of each group with HC to produce a t-map. Minimum cluster size was set to 100 voxels to minimize the effect from noise. The significance level of the group difference was threshold based on the two-tailed Gaussian Random Field (GRF) theory, with voxel level of *p* < 0.01 and cluster level of *p* < 0.05. A VMHC map was obtained. A location with peak t-value was recorded as region of interest (ROI). Brain regional volume and leukoaraiosis volume between different groups were examined using MANCOVA test, using age, gender, and total intracranial volume as covariates. To investigate whether the IFC was affected by the volume, we studied the relationship between the VMHC values and their corresponding brain regional volumes using partial correlation analysis, with age and sex as covariates. 

To access the diagnostic efficacy of neuronal spontaneous activity, the VMHC value of brain regions derived based on AAL (anatomical automatic labeling) template were extracted using REST software version 1.8. The predictive performance of VMHC values was calculated using Statistical Product and Service Solutions (SPSS) Ver25.0 based on the results of specificity, sensitivity, and area under the receiver operating characteristics (ROC) curves (AUC). The AUC values were graded i.e., 0.7–0.8 as fair, 0.8–0.9 as good, and 0.9–1 as excellent [40]. Optimal threshold in every brain region was determined by maximizing the Youden’s index (sensitivity + specificity −1) and accuracy. To combine the effect of altered VMHC brain regions, binary logistic regression analysis was conducted using SPSS version 25.0. The statistical significance was set as *p* < 0.05.

## 3. Result

### 3.1. Demographics, Clinical and Neuropsychological Assessments

Findings on demographics, clinical and neuropsychological test scores (HK-MoCA) of VD, AD, MCI, and HC are listed in Table 1. The age of VD and MCI was significantly higher than that of HC with *p* < 0.05 and *p* < 0.01, respectively. The HK-MoCA scores in AD were significantly lower than that of the other groups, and all diseased groups were significantly lower than that of HC. The leukoaraiosis volume in VD was significantly larger than the other three groups. Additionally, a higher percentage of participants in the VD group had T2DM, hypertension and hyperlipidemia when compared to other groups. 

### 3.2. VMHC Map in VD, AD and MCI Compared to HC

**Figure 1:**
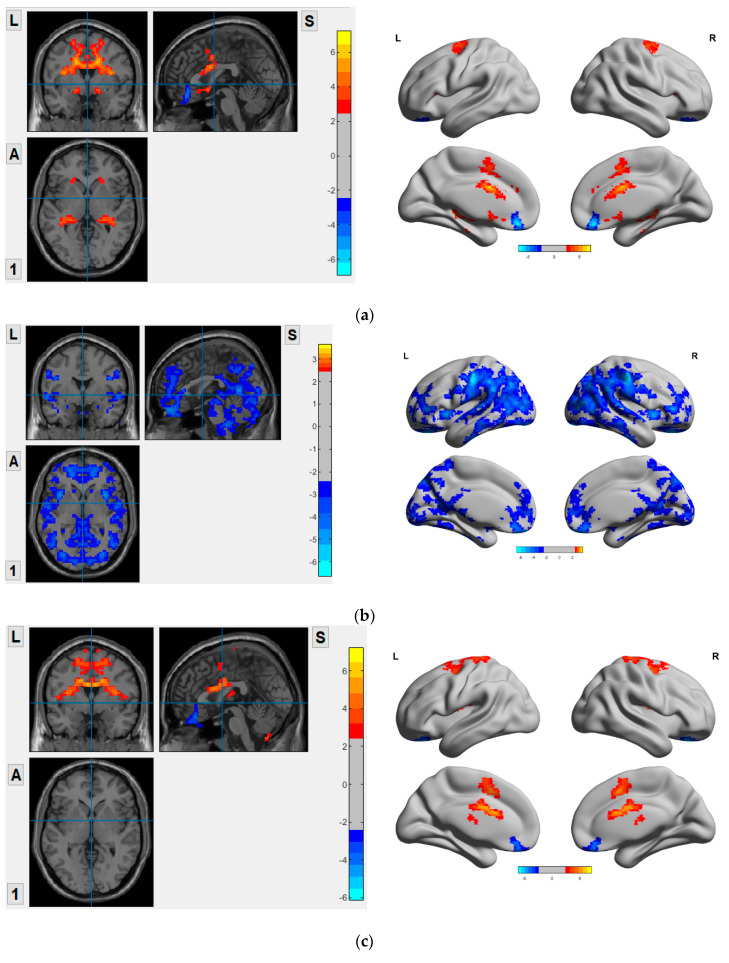
VMHC map in VD, AD, and MCI compared to HC. (**a**) VD vs. HC; (**b**) AD vs. HC; (**c**) MCI vs. HC. L: Left; S: Superior; A: Anterior; 1: Posterior.

The two-sample independent t-test VMHC maps of diseased groups as compared to HC in both global and regional levels are presented in Figure 1, i.e., VD vs. HC, AD vs. HC, and MCI vs. HC. Compared to the HC, VD showed significant reduction in VMHC in superior and medial frontal orbital gyrus, gyrus rectus. Increased VMHC were observed in superior frontal gyrus, inferior frontal gyrus operculum and triangular, precentral gyrus, rolandic operculum, supplementary motor area, insula, anterior, middle and posterior cingulate, hippocampus, parahippocampus, amygdala and caudate when compared to HC (*p* < 0.05 GRF corrected), as shown in Figure 1a. AD showed significantly decreased VMHC in the DMN, ECN, and SN (*p* < 0.05 GRF corrected), as shown in Figure 1b. MCI showed significant VMHC reduction in superior, medial, and inferior frontal orbital gyrus, olfactory and gyrus rectus. Increased VMHC were observed in supplementary motor area, precentral gyrus, postcentral gyrus, rolandic operculum, insula, middle cingulate, paracentral lobule, caudate, putamen and thalamus when compared to HC (*p* < 0.05 GRF corrected), as shown in Figure 1c. 

### 3.3. VMHC Analysis 

From the above maps, brain regions with significant different VMHC values (T value) between groups were obtained. The results are listed in Table 2, Table 3 and Table 4. Negative intensity indicates lower VMHC values of the diseased group when compared to HC. Negative volume indicates regional volume reduction when compared to HC.

Table 2, Table 3 and Table 4. Brain regions that showed significant differences in VMHC values when compared to HC. All cluster size >100 voxels, adjusted for age, gender, and total intracranial volume. # indicated significant volume change between the two groups. 

### 3.4. Correlation between VMHC Values and Their Corresponding Brain Regional Volumes

Compared the cognitive impaired groups with HC, the regions of interest with significant volume reduction (*p* < 0.05) are listed in Table 2, Table 3 and Table 4. In VD, hippocampus and amygdala showed atrophy, but there was no significant correlation between VMHC values and their corresponding volumes. In AD, more regions showed atrophy when compared to VD, but only middle occipital gyrus showed a negative correlation r = 0.692 with VMHC values. In MCI, there was no significant correlation between VMHC values and their corresponding volumes. In conclusion, the variations in VMHC values were not related to the volume variations in all studied groups. 

### 3.5. Diagnostic Accuracy of VMHC in Cognitive Impaired Groups

#### 3.5.1. ROC for VD vs. HC

**Figure 2:**
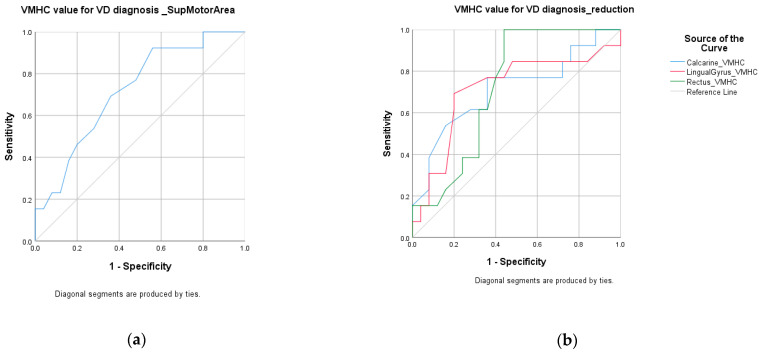
ROC curve: VD vs. HC group. (**a**) VD vs. HC group—increased VMHC; (**b**) VD vs. HC group—reduced VMHC.

**Figure 3:**
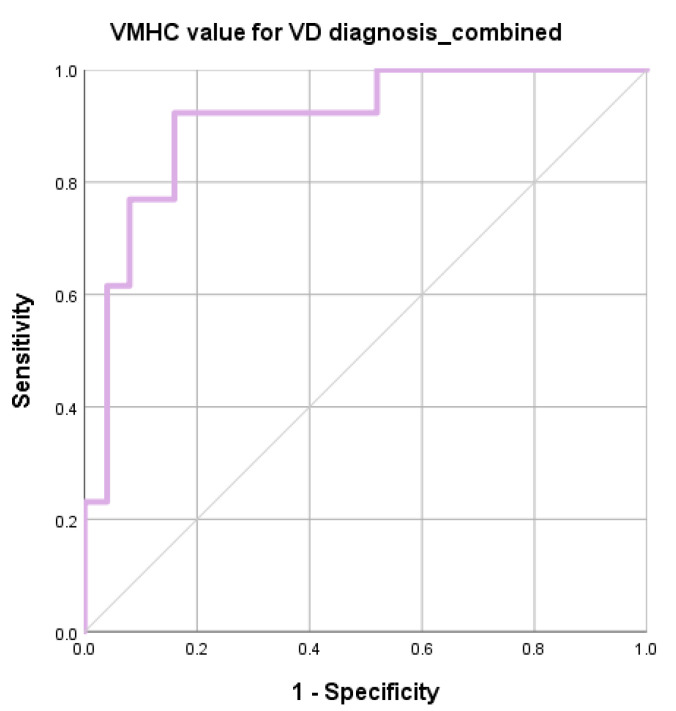
ROC curve: VD vs. HC group: combined regions.

ROC analysis demonstrated that the regional VMHC changes exhibit fair performance in distinguishing VD patients from HC. Reduced VMHC was found in calcarine (accuracy = 70%, *p* = 0.034), lingual gyrus (accuracy = 75%, *p* = 0.034), and gyrus rectus (accuracy = 78%, *p* = 0.026). Increased VMHC was found at Supplementary motor area (accuracy = 68%, *p* = 0.029). Combining the VMHC values of these four regions, the accuracy improved to 87% with AUC over 0.9. Balanced sensitivity and specificity were achieved. Details are listed in Table 5. The ROC curves are shown in Figure 2 and Figure 3.

#### 3.5.2. ROC for AD vs. HC

**Figure 4:**
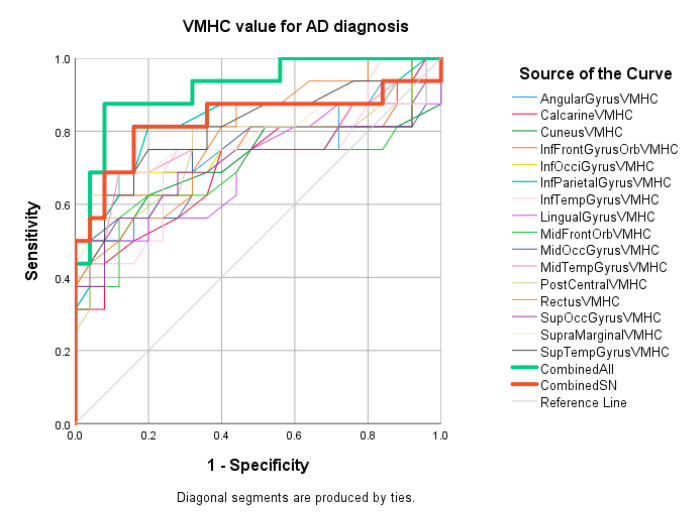
ROC curve: AD vs, HC group.

ROC analysis demonstrated that the regional VMHC changes of inferior parietal (accuracy = 81%, *p* = 0.001), superior temporal gyrus (accuracy = 80%, *p* = 0.001), and inferior occipital gyrus (accuracy = 80%, *p* = 0.01) exhibited good performance in distinguishing AD patients from HC. Balanced sensitivity and specificity were achieved in these three regions. The other 12 regions showed fair accuracy in the ROC analysis. After combining the VMHC values of all 15 regions, the accuracy improved to 92% with AUC over 0.9. Details are listed in Table 6. The ROC curves are shown in Figure 4.

#### 3.5.3. ROC for MCI vs. HC

**Figure 5:**
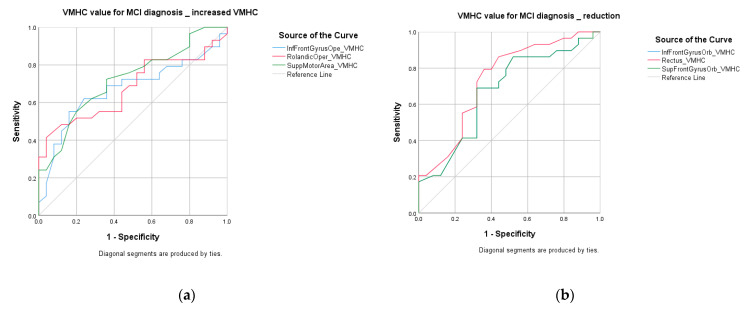
ROC curve: MCI vs. HC group. (**a**) MCI vs. HC: increased VMHC; (**b**) MCI vs. HC: reduced VMHC.

**Figure 6:**
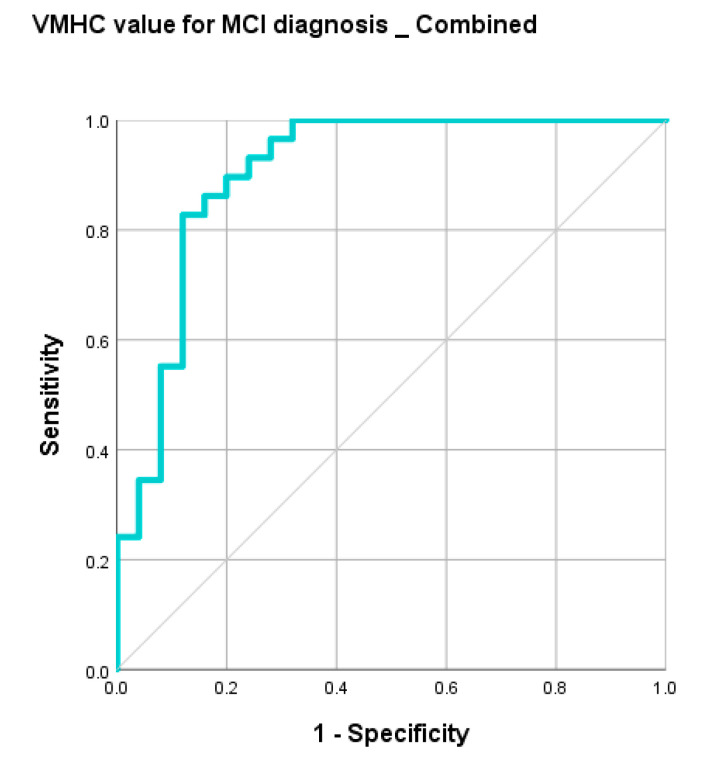
ROC curve: MCI vs. HC group: combined regions.

ROC analysis demonstrated that regional VMHC changes exhibit fair performance in distinguishing MCI from HC. Activated VMHC was found in inferior frontal operculum (accuracy = 70%, *p* = 0.031), Rolandic operculum (accuracy = 68%, *p* = 0.026), and supplementary motor area (accuracy = 68%, *p* = 0.006); attenuated VMHC was found in inferior frontal orbital gyrus (accuracy = 68%, *p* = 0.031), gyrus rectus (accuracy = 72%, *p* = 0.003), and superior frontal orbital gyrus (accuracy = 68%, *p* = 0.03). After combining the VMHC values of inferior frontal operculum, Rolandic operculum, supplementary motor area, inferior frontal orbital gyrus, and gyrus rectus, the accuracy improved to 83% with AUC over 0.9. Balanced sensitivity and specificity were achieved. Details are listed in Table 7. The ROC curves are shown in Figure 5 and Figure 6.

## 4. Discussion

Our study investigated rs-fMRI IFC in VD, AD, and MCI, and produced three main findings: first, VD, similar to AD and MCI, showed aberrant VMHC when compared to HC. Secondly, the aberrant VMHC was independent to its corresponding brain regional volume but may relate to the leukoaraiosis and vascular risk factors in VD, and related to amyloid burden in AD and MCI. Thirdly, combining regional VMHC achieved high accuracy to differentiate VD, AD, and MCI from HC, with balanced sensitivity and specificity in ROC analysis. Thus, VMHC has the potential to be a good diagnostic tool in characterizing the three cognitive impaired groups from HC.

### 4.1. VMHC and Brain Regional Volume Change Are Two Independent Metrics

Our results demonstrated that brain regional volume reduction was independent to the variation of VMHC values. The results were coherent with our expectations. VMHC measured IFC was affected in the early stage of dementia; whereas, structural changes would usually exist in the later stage of the disease. The relationship between VMHC and brain regional volume was weak and indirect.

### 4.2. Aberrant VMHC in the Cognitive Impaired Groups and Its Diagnostic Accuracy

Interhemispheric coordination impairment is a classical deficit of patients suffering from dementia. Compare to structural imaging, it is a sensitive biomarker which is useful in early diagnosis. To study interhemispheric coordination, EEG was used by Duffy and his team [41]. Until recently, functional MRI was implemented in AD and MCI diagnosis, which allowed voxel-wised whole brain study [23,24,25]. It helped clinicians to differentiate AD and MCI from HC. VD diagnosis still mainly relies on structural imaging, such as FLAIR and DW MRI. The application of fMRI in VD was mainly on priori based graph theoretical analysis [42], seed based analysis [43], or using particular band frequency [44]. The IFC analysis in VD was limited. Therefore, the aberrant VMHC maps presented in this study, together with the result in ROC analysis could be a potential biomarker to supplement the early diagnosis of VD.

#### 4.2.1. VD vs. HC

Our result showed that IFC decreased in frontal orbital gyrus and gyrus rectus, but increased IFC in DMN including superior frontal gyrus, inferior frontal gyrus triangular, anterior and posterior cingulate gyrus; in precentral gyrus of ECN; in other parts of SN including amygdala, insula, inferior frontal operculum, and rolandic operculum; in the supplementary motor area. 

Previous studies suggested that vascular cognitive impairment encompasses all causes of cerebrovascular diseases, including silent brain infarct, leukoaraiosis, lacunar infarcts, medial temporal atrophy, and vascular comorbidities [12,45]. In this study, VD showed a significantly larger volume of leukoaraiosis, medial temporal atrophy, and higher vascular comorbidities when compared to the other three groups. 

Leukoaraiosis has been reported as having a strong association with global cognition, executive function, and psychomotor response [46]. VD in this study demonstrated severe leukoaraiosis with significantly lowered HK-MoCA score compared to HC. Chen et al. (2019), demonstrated that severe leukoaraiosis associated positively with enhanced IFC within SN [47]. The anterior insula and anterior cingulate gyrus plays an important role in SN, which is modulated by homeostatic, cognitive, and emotional stimuli [48,49], and as a switch between ECN and the DMN across different stimulus modalities. Enhanced IFC in SN can be a compensatory action to maintain the regulation between ECN and DMN, which are vital to maintain daily activities. However, Chen’s study used ICA to interpret functional connectivity, which required a priori region of interest for analysis. VMHC is a voxel-wise study of whole brain study of functional connectivity, all voxels were analyzed and provided comprehensive maps for comparison. 

Additionally, our result demonstrated IFC decreased in frontal orbital gyrus, which encompass many different functions, including sensory integration to modulate behavior through the motor system, learning participation, and decision making for reward related behavior [50]. A previous study described that patients with dysfunction in frontal orbital cortex had impulsive responses, including deficits in motor response inhibition [51]. This may explain the increased IFC in the supplementary motor area to compensate for such deficits. The study of Liu et al. (2015) suggested that the IFC increased in bilateral motor cortex was to compensate the anatomical connection damage caused by the subcortical stroke [52]. Further investigation is needed to identify the correlation between the two regions. 

Medial temporal atrophy represents volume loss in the hippocampal region [53]. It is one of the sensitive biomarkers identified for AD and associated cognitive impairment [54] and VD [55]. Our study result was similar to the Cho et al. (2009) study, which indicated the atrophy in AD was larger than that in VD. However, atrophy presents in later stage as the disease develops. For early diagnosis, upstream imaging techniques such as functional MRI are recommended as supplementary investigation.

In view of vascular comorbidities, the study of Schuff et al. (2009) suggested that the mentioned risk factors (T2DM, hypertension, and hyperlipidemia) were associated with cerebral perfusion decline [56]. Their study showed that both gray matter atrophy and cerebral blood flow (CBF) reduction coexist in frontal and parietal cortex in VD, similar to the location of IFC reduction in our study. It suggested that those risk factors might contribute to the IFC reduction in our cohort. Further investigation on CBF is suggested to investigate the association between IFC and CBF. In addition, Hachinski et al. (2008) suggested that cognitive impairment could result from severe microvascular brain disease [57]. The metabolic reduction showed in different anatomical regions between AD and VD, were similar to the regions of interest in our study, and might help to differentiate VD from AD.

#### 4.2.2. AD vs. HC

Compared to HC, significantly lowered VMHC was observed throughout the limbic, frontal, parietal, temporal, occipital regions, that is DMN, ECN, and SN. Disruption of the network will have a negative effect on normal functioning and caused severe consequences. Our study showed similar results to the study of Wang et al. (2015), where they found that VMHC decreased in the orbital frontal cortex, anterior cingulate, putamen, caudate, insula nucleus accumbens, and primary olfactory cortex [24]. According to the findings in Braak and Braak 1997, Wang’s study matched the definition of amyloid deposits during the intermediate stage of dementia (between stages A and B) [24,58]. The amyloid deposits were found in basal neocortex, including perihinal and ectorhinal regions initially. The depositions increase and spread to olfactory, anterior cingulate, orbitofrontal, caudate, insula, and hippocampal regions at stage B. Our result showed reduced VMHC in a more widely spread manner, in operculum, superior frontal gyrus, middle temporal gyrus, fusiform gyrus, inferior temporal gyrus, lingual gyrus, calcarine, cuneus, and occipital lobe at intermediate stage (stage B); also advanced stage (stage C) including posterior cingulate, precuneus, and paracentral gyrus (parietal lobe). It matched the definition of advanced stage of amyloid deposition (stage C). Results from both studies matched the description of amyloid deposition pattern described in the study of Braak and Braak (1997). It suggested that interhemispheric functional connectivity decreases with the accumulation of amyloid in AD. 

In view of using VMHC value as a diagnostic test, Yang et al. (2018) used amplitude of low frequency fluctuations (ALFF) and fractional ALFF (fALFF) to differentiate AD from HC, They achieved an accuracy of 80.2% for AD vs. HC and 75.36% for MCI vs. HC using ALFF based ROC analysis; while using fALFF features, they achieved an accuracy of 89.1% and 70%, respectively, in ROC analysis [59]. Our results achieved accuracy that ranged from 72% to 81% using regional VMHC (81% for AD vs. HC, 72% for MCI vs. HC, and 78% for VD vs. HC). Our results were comparable to theirs even though we used a different method of analysis. In contrast to our study, they incorporated ALFF or fALFF values of 116 brain regions divided based on anatomical automatic labeling (AAL) template, and we combined regional VMHC where they were significantly different from HC. We both yielded higher accuracy when combining functional connectivity of different regions, indicating that functional connectivity of multiple regions improved the diagnostic test capability. 

#### 4.2.3. MCI vs. HC

MCI is a precursor to AD, and it has been classified as the prodromal stage of Alzheimer’s disease [60]. In the current study, the IFC reduction was only confined to the anterior brain regions (gyrus rectus, olfactory, superior and inferior frontal orbital gyrus). Wang et al. (2015) showed a similar pattern of decreased VMHC in anterior brain regions including orbitofrontal, anterior cingulate, nucleus accumbens when compared to HC. However, increased VMHC was found in the sensorimotor cortex when compared to HC [24]. The anterior brain regions matched the description of early stage of amyloid deposition in the study of Braak and Braak (1997). While, the increases of VMHC in Rolandic operculum, superior frontal gyrus, insula, caudate, putamen, thalamus (amyloid spread to stage B) and precentral gyrus, paracentral gyrus, post central gyrus and supplementary motor area (amyloid spread stage C) follows the route of spread of amyloid deposition. The trend suggested that mild deposition of amyloid induces functional connectivity increases to compensate the loss of function [58]. Qureshi et al. (2019) used independent component analysis (ICA) to classify different levels of cognitive impairment and they achieved classification accuracy of 92.3% based on the rs-fMRI data. With the power of artificial intelligence, more rs-fMRI features can be included for analysis that may improve the diagnostic accuracy.

Other than using resting state functional MRI to study functional connectivity in detection of AD, MCI vs. HC, brain structural change has been used to differentiate AD and MCI from HC. Hippocampal atrophy was used and achieved AUC 74% and 65% for AD vs. HC and MCI vs. HC comparison, respectively, in Teipei et al. (2017) [61]. However, hippocampal atrophy usually exists in the late stage of AD. As the development from MCI to AD can last for years, it is not a good diagnostic tool for early AD or MCI detection. Furthermore, our results achieved higher AUC with balanced sensitivity and specificity. It indicated that VMHC is a better tool in differentiating AD and MCI from HC compared to using hippocampal volume reduction. 

### 4.3. VMHC at Olfactory Network and Salient Network

Gyrus rectus lies medial to olfactory sulcus which located in the frontal lobe, and inferior to the olfactory frontal sulcus [62]. The olfactory network includes frontal orbital gyrus, olfactory cortex, and insula [63]. For all three diseased groups, the VMHC values in gyrus rectus and olfactory network were significantly lowered when compared to HC. A previous study showed no significant correlation between gyrus rectus and cognitive outcome in terms of MMSE, but subgroup analysis showed that language and memory recall were affected after surgical removal of gyrus rectus [64]. Lu et al. (2019) suggested that functional connectivity between the olfactory network to the right hippocampus were reduced in early stage MCI, late stage MCI and AD when compared to HC [65]. The level of impairment is directly related to the disease stage from early MCI to AD. Our result showed attenuated IFC in frontal orbital gyrus in VD but activated IFC in insula and olfactory. Insula is involved not only in the olfactory network, but also in the salient network. The function of the salient network (dorsal ACC and bilateral insula) appears to help in selection and segregation of relevant internal and external stimuli to guide behavior [66]. We hypothesized that the activated IFC in insula was related to promoting the function of the salient network to maintain daily functioning for VD patients by increased IFC in DMN and ECN.

### 4.4. Limitations and Future Study

Firstly, the sample sizes of this study in different diseased groups were small. Thus, our study result should be considered as a pilot study. Additionally, the number of participants with Lewy body dementia (DLB) and frontotemporal dementia (FTD) were too little for adequate evaluation. Secondly, due to the involvement of radiopharmaceutical injection, HC did not undergo F18-flutemetamol PET scan. Thirdly, vascular risk factors (e.g., diabetes status and lipid profile) of participants were obtained, but correlation analysis was not performed. Our current results were more exploratory and descriptive in nature. Additionally, detailed neuropsychological evaluation with impaired performance in different domains was not performed. It prevented further analysis on the correlation between regional VMHC and performance in neuropsychological domains in different diseased groups. As previously mentioned, accumulation of amyloid in brain regions may affect IFC and further study about the correlation of VMHC with amyloid accumulation can be conducted. This will form the pathophysiological basis of the diagnostic capabilities of VMHC to differentiate between AD, MCI, and HC. As discussed, cerebral blood flow, macro- and micro-vascular complications, and leukoaraiosis may be risk factors for VD. Combining VMHC and the above parameters may help to improve the accuracy to differentiate VD from other types of dementia. However, the duration and medication history of T2DM, hypertension, hyperlipidemia were not collected, which limited the analysis of severity level of abovementioned vascular comorbidities. Further studies performing correlation analysis of VMHC with CBF and the status of vascular comorbidities were suggested. 

## 5. Conclusions

Our results showed that aberrant IFC was found in VD, AD, and MCI when compared to HC. VMHC maps showed specific patterns of reduction and increase of IFC in different dementia groups. Retrieving VMHC values in different brain regions can characterize AD, MCI, and VD from HC. Through combining the VMHC values at relevant brain regions, the diagnostic capabilities of AD, MCI, and VD from HC could be improved with accuracy of 92%, 83%, and 87%, respectively.

## Figures and Tables

**Table 1:** Demographics of participants.

	VD	AD	MCI	HC
No of participants	13	16	29	25
Age	79.15 ^^^ ± 4.06 (69–84)	74.81 ± 7.93 (61–87)	74.95 ^@^ ± 6.84 (64–88)	68.84 ^@^^ ± 6.27 (60–84)
Gender (M/F)	9/6	8/13	7/14	9/16
HK-MoCA	17.00 ^%^* ± 4.69 (7–23)	13.17 ^#%^* ± 7.11 (3–23)	21.25 ^#%^ ± 3.88 (13–29)	28.56 ^%^± 1.23 (26–30)
leukoaraiosis volume (mm^3^)	15,098.7 ^^^	4883.3 ^^^	3309.2 ^^^	4247.4 ^^^
T2DM	3(23.1%)	0	6 (20.6%)	0
Hypertension	9(69.2%)	7(43.7%)	13(44.8%)	7(28%)
Hyperlipidemia	10(76.9%)	6(37.5%)	4(13.8%)	4(16%)

* *p* < 0.05 ANOVA on ranks with Bonferroni post hoc test. *#@ *p* < 0.05 ANOVA on ranks with Bonferroni post hoc test. ^% *p* < 0.001 ANOVA on ranks with Bonferroni post hoc test.

**Table 2:** VD vs. HC group.

VD vs. HC Brain Regions	Network	Coordinate (MNI)			Peak Intensity	Volume	Correlation Coefficient
		x	y	z	(T Value)	(mm^3^)	
Precentral gryus	ECN	−23	−14	66	3.011	−316.38	
Sup Frontal	DMN	−19	−8	74	3.437	603.33	r = 0.667
Sup Frontal Orb	vSN	−18	38	−20	−2.711	61.50	
Inf Frontal Oper	vSN	−44	15	11	3.464	26.45	
Inf Frontal Tri	DMN	−33	29	4	2.857	−59.58	
Rolandic Oper	dSN	−40	−13	21	3.652	211.16	
Supp Motor Area		−6	7	51	3.835	−132.8	
Olfactory		−12	15	17	3.553	−69.98	
Frontal Med Orb		−6	38	−10	−4.049	−150.01	
Gyrus rectus	vSN	−6	39	−18	−6.866	−4.40	
Insula	SN	−36	−14	13	4.682	−57.48	
Ant cing	DMN	−5	11	27	4.315	−165.48	
Mid Cing	DMN	−8	8	42	3.201	−147.7	
Post Cing	DMN	−12	−43	16	2.963	−85.80	
Hippocampus		−28	−20	−10	3.391	−720.79 ^#^	
Parahippocampus		−22	−34	−5	3.141	−165.67	
Amygdala	SN	−18	−1	−11	3.707	−385.47 ^#^	
Caudate		−15	23	9	3.553	118.27	

# indicated significant volume change between the two groups.

**Table 3:** AD vs. HC group.

AD vs. HC Brain Regions	Network	Coordinate (MNI)			Peak Intensity	Volume	Correlation Coefficient
		x	y	z	(T Value)	(mm^3^)	
Precentral gryus	ECN	−40	−21	63	−2.848	−191.13	
Sup Frontal	DMN	−19	55	27	−2.574	−820.20^#^	
Sup Frontal Orb	vSN	−18	34	−24	−5.252	−81.60	
Mid Frontal	DMN	−34	52	16	−2.807	−797.45	
Mid Front Orb	ECN	−32	57	−8	−2.915	−241.39	
Inf Frontal Oper	vSN	−52	9	25	−4.033	−303.90	
Inf Frontal Tri	DMN	−45	39	10	−4.157	−291.70	
Inf Frontal orb	vSN	−37	29	−21	−3.362	−29.40	
Rolandic Oper	dSN	−45	−26	18	−2.766	−136.20	
Olfactory		−6	23	−8	−2.651	−72.54	
Sup Frontal Medial		−6	51	27	−2.852	−533.60	
Frontal Med Orb		−6	39	−9	−3.159	−328.71	
Gyrus rectus	vSN	−8	37	−19	−4.997	−57.56	
Insula	SN	−36	7	3	−3.783	−404.75	
Ant cing	DMN	−7	47	12	−3.052	−271.20	
Post Cing	DMN	−6	−43	12	−3.14	−245.20 ^#^	
Hippocampus		−22	−37	9	−2.839	−903.70 ^#^	
Parahippocampus		−22	−5	−32	−3.283	−407.66	
Amygdala	SN	−24	2	−17	−3.465	−567.81 ^#^	
Calcarine		−8	−63	16	−3.063	−299.00	
Cuneus		−8	−83	24	−3.054	−139.40	
Lingual Gyrus	SN	−17	−66	4	−3.236	−430.46 ^#^	
Sup Occipital	SN	−18	−84	28	−3.217	−238.40	
Mid occipital	ECN	−35	−81	35	−3.726	−714.76 ^#^	r = 0.692
Inf occipital		−37	−83	−4	−3.42	−139.90	
Fusiform		−28	−50	−12	−2.989	−744.70 ^#^	
Post Central	ECN	−44	−23	49	−4.312	−333.39	
Sup Parietal		−24	−59	56	−3.178	−298.60 ^#^	
Inf Parietal	DMN	−44	−36	39	−5.332	−298.56	
SupraMarginal	ECN	−57	−44	31	−5.199	−690.12	
Angular	DMN	−50	−61	29	−3.166	−603.50	
Precuneus	DMN	−3	−55	41	−3.250	−893.74 ^#^	
Caudate		−12	22	−5	−3.411	−243.47	
Thalamus		−10	−22	3	−3.059	−700.16 ^#^	
Heschl		−50	−15	9	−3.824	−9.20	
Sup Temp	DMN	−54	−6	−2	−3.853	−1304.4 ^#^	
Sup Temp Pole		−51	7	−6	−3.311	−378.75	
Mid Temp	ECN	−57	−49	6	−3.105	−834.24	
Inf Temp	ECN	−51	−51	−23	−3.535	−1240.70 ^#^	

# indicated significant volume change between the two groups.

**Table 4:** MCI vs. HC group.

MCI vs. HC Brain Regions	Network	Coordinate (MNI)			Peak Intensity	Volume	Correlation Coefficient
		x	y	z	(T Value)	(mm^3^)	
Precentral gryus	ECN	−31	−5	50	2.728	+65.00	
Sup Frontal	DMN	−19	1	59	3.839	262.16	
p Frontal Orb	vSN	−17	33	−23	−4.496	−67.40	
Inf Frontal orb	vSN	−37	29	−21	−3.362	−53.90	
Rolandic Oper	dSN	−43	−8	18	3.420	29.48	
Supp Motor Area		−8	8	46	5.041	−192.20	
Olfactory		−9	18	−4	−2.931	−43.48	
Frontal Med Orb		−6	37	−10	−2.642	−197.96	
Gyrus rectus	vSN	−8	39	−20	−4.386	54.37	
Insula	SN	−33	7	12	3.308	−101.8	
Mid cing	DMN	−8	4	43	3.734	0.40	
Post Central	ECN	−21	−30	71	3.663	−133.1	
Paracentral Lobule		−14	−26	76	3.567	22.34	
Hippocampus		No sig				−470.56 ^#^	
Amygdala	SN	No sig				−299.84 ^#^	
Caudate		−16	11	20	4.122	35.72	
Putamen		−24	8	10	2.825	−247.9	
Thalamus		−12	−14	18	2.954	−386.58 ^#^	

# indicated significant volume change between the two groups.

**Table 5:** ROC curve result for VD vs. HC group.

Brain Regions	AUC	Sig	Network		Threshold VMHC Value	Sen	Spec	PPV	NPV	Acc	Youden Index
Calcarine	0.712	*p* = 0.034	DMN	Reduction	0.47	0.77	0.64	0.68	0.73	70%	0.41
Lingual Gyrus	0.712	*p* = 0.034	dSN	Reduction	0.52	0.69	0.80	0.78	0.72	75%	0.49
Gyrus Rectus	0.723	*p* = 0.026	vSN	Reduction	0.42	1.00	0.56	0.69	1.00	78%	0.56
Supp Motor Area	0.718	*p* = 0.029		Increased	0.42	0.85	0.52	0.64	0.77	68%	0.37
Combined	0.908	*p* = 0.000				0.923	0.84	0.92	0.77	87%	0.76

**Table 6:** ROC curve result for AD-HC group.

Brain Regions	AUC	Sig	Network	Threshold VMHC Value	Sensitivity	Specificity	PPV	NPV	Accuracy	Youden Index
Angular Gyrus	0.735	*p* = 0.012	DMN	0.47	0.69	0.76	0.74	0.71	72%	0.45
Calcarine	0.709	*p* = 0.026	DMN	0.47	0.75	0.64	0.68	0.72	70%	0.39
Cuneus	0.716	*p* = 0.021	DMN	0.44	0.50	0.96	0.93	0.66	73%	0.46
Inf Parietal	0.82	*p* = 0.001	DMN	0.42	0.81	0.80	0.80	0.81	81%	0.61
Sup Temp	0.708	*p* = 0.001	DMN	0.37	0.75	0.84	0.82	0.77	80%	0.59
Inf Temp	0.73	*p* = 0.014	ECN	0.32	0.75	0.72	0.73	0.74	74%	0.47
Mid Occi	0.725	*p* = 0.016	ECN	0.33	0.50	0.96	0.93	0.66	73%	0.46
Mid Temp	0.755	*p* = 0.006	ECN	0.29	0.69	0.88	0.85	0.74	78%	0.57
Post Central	0.747	*p* = 0.008	ECN	0.29	0.56	0.92	0.88	0.68	74%	0.48
SupraMarginal	0.781	*p* = 0.003	ECN	0.29	0.63	0.96	0.94	0.72	79%	0.59
Inf Front Orb	0.754	*p* = 0.007	SN	0.29	0.63	0.88	0.84	0.70	75%	0.51
Inf Occi	0.741	*p* = 0.010	SN	0.37	0.69	0.92	0.90	0.75	80%	0.61
Lingual Gyrus	0.7	*p* = 0.033	SN	0.45	0.50	0.96	0.93	0.66	73%	0.46
Gyrus rectus	0.787	*p* = 0.002	vSN	0.32	0.56	0.88	0.82	0.67	72%	0.44
Sup Occi	0.708	*p* = 0.002	SN	0.38	0.56	0.88	0.82	0.67	72%	0.44
Combined SN	0.83	*p* = 0.000			0.81	0.84	0.63	0.96	83%	0.65
Combined all	0.92	*p* = 0.000			0.92	0.88	0.88	0.92	92%	0.76

**Table 7:** ROC curve result for MCI vs. HC group.

Brain Regions	AUC	Sig	Network		Threshold VMHC Value	Sens	Spec	PPV	NPV	Acc	Youden Index
Inf Front Oper	0.671	*p* = 0.031	vSN	Increased	0.4	0.55	0.84	0.78	0.65	70%	0.39
Rolandic Oper	0.677	*p* = 0.026	dSN	Increased	0.46	0.48	0.88	0.80	0.63	68%	0.36
Supp Motor Area	0.721	*p* = 0.006		Increased	0.47	0.52	0.84	0.76	0.64	68%	0.36
Inf Front Orb	0.672	*p* = 0.031	vSN	Reduction	0.35	0.69	0.68	0.68	0.69	68%	0.37
Gyrus rectus	0.736	*p* = 0.003	vSN	Reduction	0.39	0.76	0.68	0.70	0.74	72%	0.44
Sup Front Orb	0.672	*p* = 0.03	vSN	Reduction	0.35	0.69	0.68	0.68	0.69	68%	0.37
Combined	0.905	*p* = 0.000				0.88	0.83	0.80	0.86	83%	0.71

## Data Availability

The clinical data and MRI images are not publicly available for patient privacy protection purposes.
